# Autophagy inhibition sensitizes hepatocellular carcinoma to the multikinase inhibitor linifanib

**DOI:** 10.1038/srep06683

**Published:** 2014-10-20

**Authors:** Hongming Pan, Zhanggui Wang, Liming Jiang, Xinbing Sui, Liangkun You, Jiawei Shou, Zhao Jing, Jiansheng Xie, Weiting Ge, Xiujun Cai, Wendong Huang, Weidong Han

**Affiliations:** 1 Department of Medical Oncology, Sir Run Run Shaw Hospital, College of Medicine, Zhejiang University, Hangzhou, Zhejiang, China; 2 Laboratory of Cancer Biology; Institute of Clinical Science, Sir Run Run Shaw Hospital, College of Medicine, Zhejiang University, Hangzhou, Zhejiang, China; 3 Cancer Institute, The Second Affiliated Hospital, College of Medicine, Zhejiang University, Hangzhou, Zhejiang, China; 4 Department of General Surgery, Sir Run Run Shaw Hospital, College of Medicine, Zhejiang University, Hangzhou, Zhejiang, China; 5 Division of Molecular Diabetes Research, Department of Diabetes and Metabolic Diseases Research, Beckman Research Institute, City of Hope National Medical Center, Duarte, CA

## Abstract

Autophagy is a critical survival pathway for cancer cells under conditions of stress. Thus, induction of autophagy has emerged as a drug resistance mechanism. This study is to determine whether autophagy is activated by a novel multikinase inhibitor linifanib, thereby impairing the sensitivity of hepatocellular carcinoma (HCC) cells to this targeted therapy. Here, we found that linifanib induced a high level of autophagy in HCC cells, which was accompanied by suppression of phosphorylation of PDGFR-β and its downstream Akt/mTOR and Mek/Erk signaling pathways. Cell death induced by linifanib was greatly enhanced after autophagy inhibition by the pharmacological inhibitors or siRNAs against autophagy related genes, ATG5 and ATG7, *in vitro.* Moreover, HCQ, an FDA-approved drug used to inhibit autophagy, could significantly augment the anti-HCC effect of linifanib in a mouse xenograft model. In conclusion, linifanib can induce cytoprotective autophagy by suppression of PDGFR-β activities in HCC cells. Thus, autophagy inhibition represents a promising approach to improve the efficacy of linifanib in the treatment of HCC patients.

Hepatocellular carcinoma (HCC) is the fifth most common cancer in the world and the third cause of cancer mortality. Surgical resection or liver transplantation is the main therapeutic strategy for patients at early stages^1^. However, most patients reach an advanced stage for the first diagnosis of HCC, and lose the opportunity for surgical operation. Conventional chemotherapy is usually ineffective with a low response rate (about 3.3–28.7%) in patients with advanced, relapsed or unresectable HCC^2^. Sorafenib, a potent multikinase inhibitor, has been recognized as the standard systemic treatment for patients with advanced HCC based on the results from SHARP trial^3^. However, compared with placebo groups, sorafenib only improved 2.8 months in median overall survival (OS) and 2.7 months in median progression-free survival (PFS)^3^. Thus, there is still an urgent need to develop more effective therapeutics to circumvent the resistance for this aggressive cancer.

Linifanib is a structurally novel and potent inhibitor of members of the vascular endothelial growth factor (VEGF) and platelet-derived growth factor (PDGF) receptor families^4^. PDGF belongs to the cystine containing protein superfamily of structurally and functionally related signaling molecules, which includes four PDGF growth factors (PDGF-A, PDGF-B, PDGF-C and PDGF-D)^5^. These ligands exert their cellular effects via their homologous receptors PDGFR-α and PDGFR-β, which are isomers belonging to the class III receptor tyrosine kinase (RTK) family. Binding to the PDGFR, the ligands induce the dimerization of PDGFR and autophosphorylation; subsequently activate its intrinsic tyrosine kinase activity. Intracellular phosphotyrosine recruits Src homology 2 (SH2) domain-containing molecules, then initiate the activation of various signaling pathways, including the Ras-Mek-Erk pathway, the PI3K-Akt pathway, the PLC-γ pathway, and the Src pathway^6^,^7^. These signaling pathways ultimately lead to cellular proliferation, differentiation, survival and migration. Linifanib has extensive anti-cancer activities in various solid tumors, including small-cell lung carcinoma, colon carcinoma, breast carcinoma and Ewing sarcoma *in vitro* and *in vivo*^4^,^8^. Linifanib also has synergistic anti-proliferation effects on acute myeloid leukemia in combination with Ara-c^9^. A phase II clinical trial in patients with unresectable or metastatic HCC demonstrated that linifanib had a respectable anti-HCC efficacy with a median PFS of 3.7 months, and a median OS of 9.7 months (10.4 months for patients with Child-Pugh class A hepatic function)^10^. However, an open-label, randomized phase III study of the efficacy and tolerability of linifanib versus sorafenib in subjects with advanced hepatocellular carcinoma (NCT01009593, www.clinicaltrials.gov) demonstrated that linifanib was not superior to sorafenib^11^. The potential explanation is that intrinsic or acquired resistance occurs in HCC cells treated with linifanib. An understanding of the molecular mechanisms underlying resistance to linifanib may help to improve the efficacy of this multikinase inhibitor.

Autophagy is a highly conserved catabolic process that deliveries cellular macromoleculars and organelles to lysosome for degradation in eukaryotic cells. The function of autophagy is not only located in maintaining homeostatic balance, but also involved in many specific physiological and pathological processes, such as development, aging, tumorigenesis and so on^12^. Under cellular stress conditions, such as nutrient-deficiency, chemotherapy and radiation, autophagy is rapidly activated to promote the survival of tumor cells under these unfavorable conditions. Thus, autophagy was proposed as a potential mechanism of cancer drug resistance. Accumulating evidences indicate that inhibition of autophagy enhances the efficacy of numerous anti-tumor agents, such as cisplatin, doxorubicin, sorafenib, cetuximab^13^,^14^,^15^,^16^. Currently, more than 20 phase I/II clinical trials are open to evaluate the efficacy of autophagy inhibitor combined with chemo- or targeted-therapy in a variety of cancers (www.clinicaltrials.gov). In our previous study, we showed that epidermal growth factor receptor (EGFR) inhibitor gefitinib and erlotinib both induced autophagy in lung cancer cells. Inhibition of autophagy increased the sensitivity of lung cancer cells to EGFR inhibitors, suggesting a novel approach to enhance targeted therapy of lung cancer^17^. Given that autophagy plays an important role in cancer drug resistance, we ask whether autophagy can be activated by linifanib, thereby impairing the sensitivity of HCC cells to this multi-targeted inhibitor.

In the current study, we first demonstrate that linifanib activated autophagy in hepatocarcinoma cells through inhibition of PDGFR-β signaling. Blockage of autophagy enhanced the anti-HCC activity of linifanib both *in vitro* and *vivo*, suggesting a novel and promising strategy to increase the clinical efficacy of linifanib for the treatment of advanced or unresectable HCC patients.

## Results

### Linifanib induces autophagy in HCC cells

**Figure 1:**
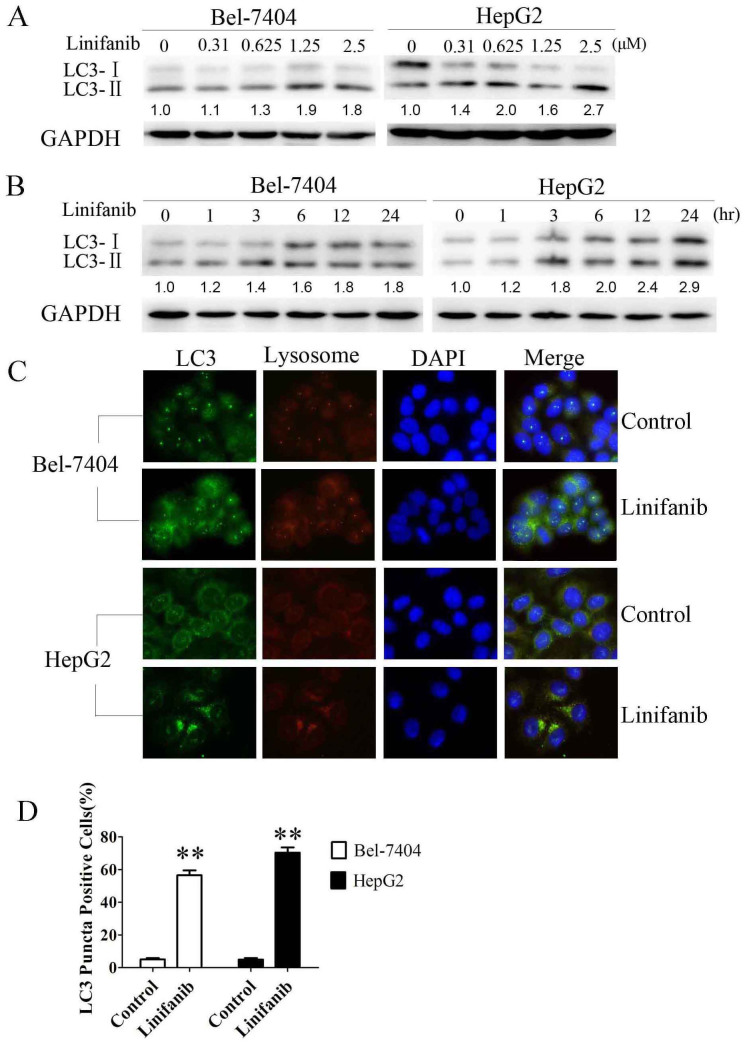
Effect of linifanib on LC3 processing. (A) & (B). Cells were incubated with the indicated concentration of linifanib for 24 h or incubated with 2.5 μM linifanib for the indicated intervals, and the transition of LC3-I to LC3-II was analyzed by Western blotting. Relative quantity of LC3-II was calculated by ImageJ densitometric analysis and normalized by GAPDH. (C). Cells were treated with DMSO or 2.5 μM linifanib for 24 h before they were labeled with fluorescence and imaged by fluorescence microscope. Green: FITC-labeled LC3; Red: lyso-tracker-labeled lysosome; Blue: DAPI-labeled nucleus. (D). Percentage of green or yellow puncta-positive cells was quantified and analyzed using a threshold of >5 dots/cell. The data were represented as the mean ± SD. **P < 0.01.

**Figure 2:**
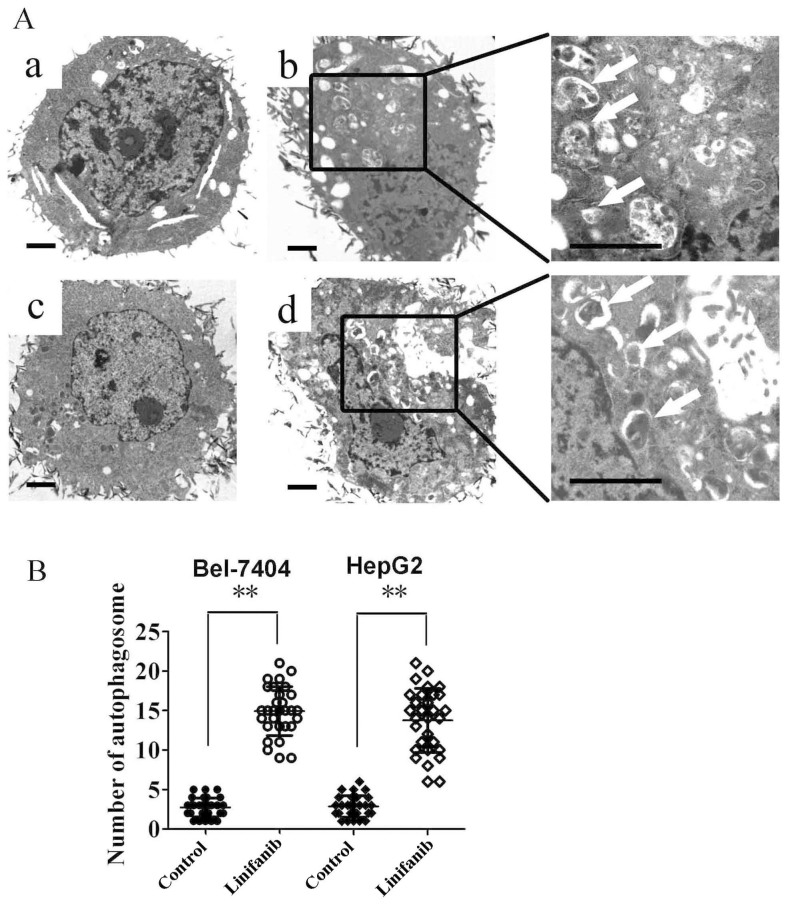
TEM depicting ultrastructures of autophagosome in cells treated with linifanib. (A). Numerous typical double-layer membrane autophagosomes (arrows) appeared in Bel-7404 and HepG2 cells treated with 2.5 μM linifanib for 24 hours. (a: Bel-7404, DMSO; b: Bel-7404, linifanib; c: HepG2, DMSO; d: HepG2, linifanib). (B). The number of autophagosomes was calculated by continuous counts in 10 fields under high resolution. Bar = 1 μm. ** P < 0.01.

To evaluate the effect of linifanib on autophagy, we examined the occurrence of autophagy in linifanib-treated HCC cells. LC3 is guided to phagophore in the process of autophagosome formation and is used as a significant marker of autophagy^18^. In human hepatoma Bel-7404 and HepG2 cells, linifanib can induce the switch of LC3-I to LC3-II in dose and time dependent manners, indicating that autophagy was activated by linifanib (Fig. 1A & B). To further confirm this observation, the compartmentalization of endogenous LC3-II in cells treated with linifanib was monitored by indirect immunofluorescence staining. Specific punctate distribution of endogenous LC3-II was observed in linifanib-treated cells. The LC3 puncta, at least partially, were co-localized with lysosomes, indicating the formation of autolysosomes (Fig. 1C & D). In addition, transmission electron microscopy (TEM) was used to check the formation of autophagosomes in linifanib-treated cells. As shown in Fig. 2, linifanib-treated cells exhibited the accumulation of large autophagic vacuoles with typical double-layer membrane containing organelle remnants, whereas only a few vacuoles were observed in control cells.

**Figure 3:**
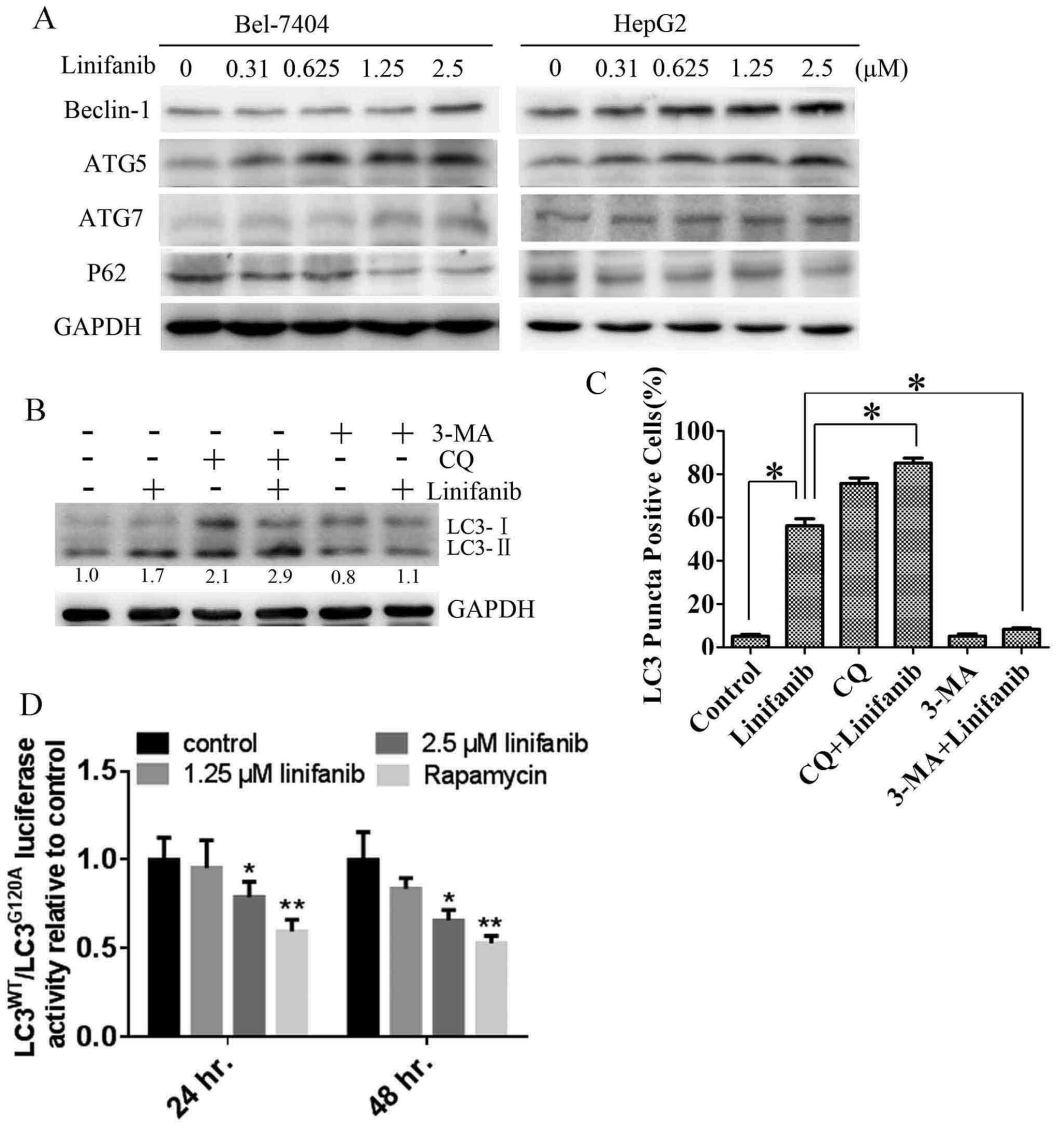
Linifanib activates autophagic flux in hepatocarcinoma cells. (A). Immunoblotting for Beclin-1, ATG5, ATG7, and p62 using lysates from cells treated with linifanib for 24 h. (B). Immunoblotting for LC3 in Bel-7404 cells treated with 2.5 μM linifanib in the presence or absence of 5 μM CQ or 5 mM 3-MA. Relative quantity of LC3-II was calculated by ImageJ densitometric analysis and normalized by GAPDH. (C). Quantification of green or yellow puncta-positive cells by using a threshold of >5 dots/cell after treatment with 2.5 μM linifanib in the presence or absence of autophagy inhibitors for 24 h. (D). Bel-7404 RLuc–LC3^WT^ and RLuc–LC3^G120A^ cells were treated with linifanib or 125 nM rapamycin. Luciferase activity was measured at 24 and 48 hours after treatment. Results shown are the mean ± SD of at least three independent experiments. *P < 0.05. ** P < 0.01.

We next examined the effects of linifanib on the expression of ATG5, ATG7 and Beclin-1, three critical components in regulating the formation of autophagosomes^19^. As shown in Fig. 3A, linifanib treatment led to upregulation of ATG5, ATG7 and Beclin-1, but downregulation of p62 in a dose dependent manner. Because p62 is selectively incorporated into autophagosomes and is specifically degraded by autophagy, thus, the total cellular expression of p62 was inversely correlated with autophagic activity^20^.

3-MA, an inhibitor of (class III) phopshatidylinositol 3-kinase, can inhibit autophagy at initial stage. In contrast, CQ, a lysosomotropic agent, can block the fusion of autophagosomes with lysosomes and inhibit autophagy at later stage^18^. Therefore, we used 3-MA and CQ to investigate linifanib-induced autophagic flux in HCC cells. As shown in Fig. 3B, linifanib treatment led to an accumulation of LC3-II in Bel-7404 cells. Inhibition of autophagy at early stage by 3-MA abolished linifanib-induced upregulation of LC3-II. However, inhibition of autophagy at later stage by CQ enhanced linifanib-induced upregulation of LC3-II. In addition, 3-MA reduced linifanib-induced formation of autophagosomes or autolysosomes, while CQ blocked the degradation of autolysosomes (Fig. 3C and Fig. S1). These results indicate that linifanib induced autophagic flux in HCC cells.

Finally, the effect of linifanib on autophagic flux was confirmed using a functional screening approach as previously reported^21^,^22^. In this system, LC3 is fused to a renilla luciferase reporter molecule forming the RLuc–LC3 fusion protein. As LC3 itself is specifically degraded by autophagy, the level of autophagy in a Bel-7404 reporter cell line stably expressing wild-type RLuc–LC3 (RLuc–LC3^WT^) can be measured in real time using an *in vivo* RLuc substrate EnduRen^TM^^22^. As a reference control, cells expressing a mutant fusion protein, RLuc–LC3^G120A^, which is unable to undergo autophagosomal localization and is not degraded by autophagy, are assayed in parallel. Thus, the autophagic flux can be evaluated as a change in the relative levels of these two fusion proteins (LC3^WT^/LC3^G120A^). As shown in Fig. 3D, the ratio of LC3^WT^/LC3^G120A^ was significantly decreased after linifanib treatment, indicating that this multikinase inhibitor accelerates autophagic flux and stimulates the degradation of LC3.

Collectively, all these results demonstrated that linifanib induced autophagy in HCC cells.

### Linifanib-induced autophagy is mediated through inhibition of PDGFR-β signaling

**Figure 4:**
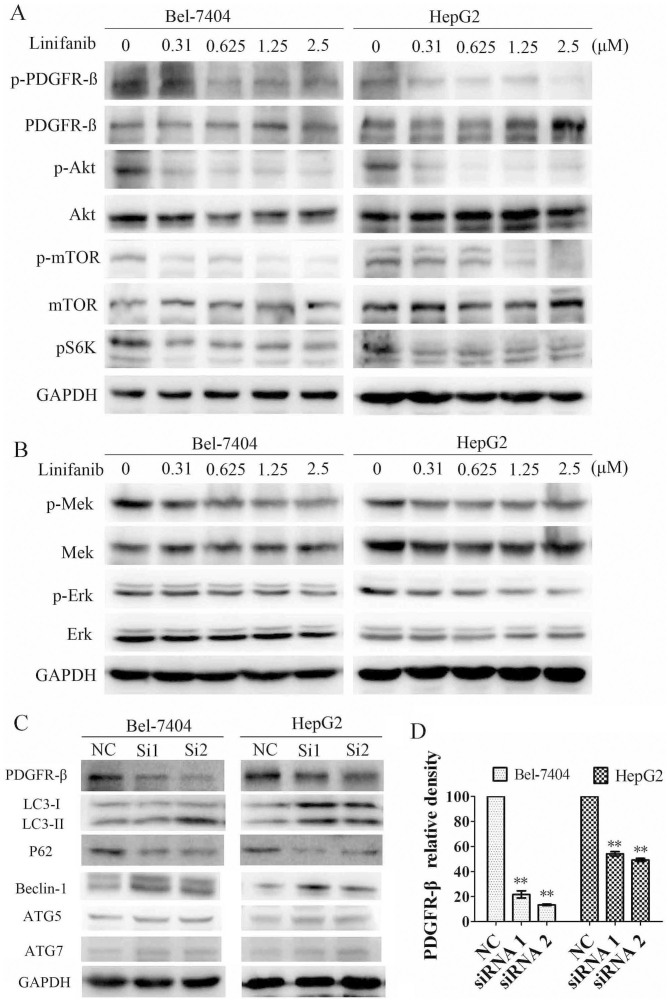
Linifanib induces autophagy by inhibiting the phosphorylation of PDGFR-ß, Akt/mTOR and Mek/Erk signaling. (A) & (B). Immunoblotting for phospho- or total PDGFR-ß, Akt, mTOR, S6K, Mek and Erk in cells treated with linifanib for 24 h. (C). Immunoblotting for LC3, p62, Beclin-1, ATG5 and ATG7 in cells transfected with siRNA against PDGFR-ß. (D). Quantification of PDGFR-ß in cells transfected with siRNAs. Data were expressed as the mean ± SD. ** P<0.01.

Linifanib is a potent and selective inhibitor of VEGFR and PDGFR^4^. Previous studies indicated that PDGFR-β was high expressed in HCC patients^23^,^24^,^25^. PI3K/AKT and Mek/Erk are two critical pathways downstream of PDGFR-β^26^. PI3K/AKT/mTOR and Ras/Raf/Mek/Erk signaling pathways are two main pathways that can regulate autophagy^27^. Given that PDGFR-β plays an important role in PI3K-AKT and MAPK pathways, we then ask whether PDGFR-β is the pivotal regulator in linifanib-induced autophagy. As expected, the phosphorylation of PDGFR-β was inhibited by linifanib in a dose-dependent manner, while the total PDGFR-β was unaffected (Fig. 4A). Accordingly, the phosphorylation of Akt, mTOR, p70S6, Mek and Erk in Bel-7404 and HepG2 cells were significantly reduced after treatment with linifanib, whereas the total levels of these proteins were unaffected (Fig. 4A & B).

To further define the roles of PDGFR-β in regulation of autophagy, two specific siRNAs were used to knockdown the expression of PDGFR-β. As show in Fig. 4 C & D, the knockdown efficiencies of these two siRNAs were 78.3 ± 4.99% and 86.6 ± 1.04% in Bel-7404 cells, and 45.9 ± 3.05% and 50.8 ± 2.12% in HepG2 cells, respectively. Knockdown of PDGFR-β resulted in upregulation of LC3-II and downregulation of p62 (Fig. 4C), indicating that inhibition of PDGFR-β could induce autophagy in HCC cells. Similar with the linifanib-induced upregulation of autophagy related genes. PDGFR-β knockdown also resulted in upregulation of ATG5, Beclin 1 and ATG7 (Fig. 4C). The results indicate that inhibition of PDGFR-β lead to upregulation of ATG related genes and then induction of autophagy.

Taken together, these results demonstrated that linifanib could activate autophagy in HCC cells through suppressing the phosphorylation of PDGFR-β and its downstream pathways.

### Inhibition of autophagy potentiates the anti-tumor effect of linifanib *in vitro*

**Figure 5:**
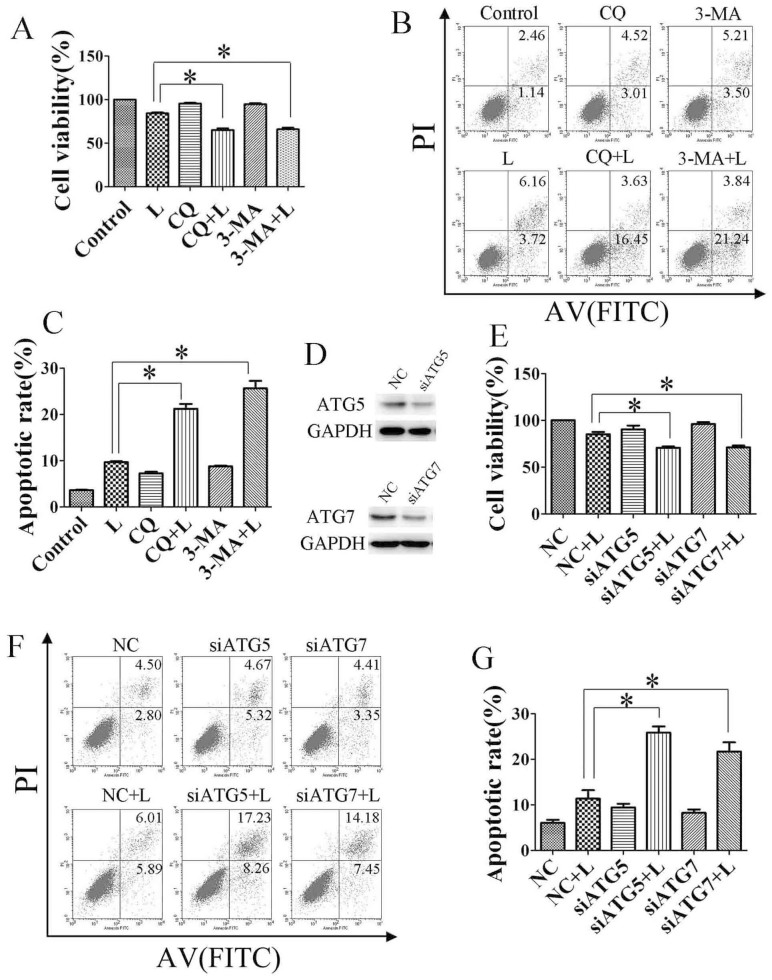
Inhibition of autophagy promotes linifanib-induced apoptosis *in vitro.* (A). Bel-7404 cells were treated with 2.5 μM linifanib (L) or DMSO in the presence or absence of CQ (5 μM) or 3-MA (5 mM) for 24 h. Cell viability was measured by MTS assay. (B) & (C). Cells were treated with linifanib alone or in combination of CQ or 3-MA before staining with annexin V (AV) and propidium iodide (PI) followed by flow cytometry. (D to G), Bel-7404 cells were transfected with siRNA against ATG5 or ATG7 for 24 h, then treated with linifanib for 24 h. Cell viability was measured by MTS assay (E), and apoptotic rates were determined by staining with annexin V and propidium iodide followed by flow cytometry (F & G). Data were expressed as the mean ± SD. * P<0.05.

Considering that autophagy may function as a stress-activated pro-survival mechanism in cancer cells^28^, we hypothesized that inhibiting autophagy could sensitize HCC cells to the linifanib treatment. Indeed, CQ and 3-MA significantly augmented growth inhibition induced by linifanib (Fig. 5A). Next, we confirmed that this autophagy inhibitor-augmented growth inhibition was mediated by promoting apoptosis. As shown in Fig. 5B & C, when cells were treated with 2.5 μM linifanib alone for 24 hours, the apoptotic rate of Bel-7404 reached 9.8 ± 0.58%. Impressively, when the cells were treated with 2.5 μM linifanib combined with CQ or 3-MA, the apoptotic rate increased to 20.1 ± 1.56% and 25.3 ± 2.31%, respectively.

To exclude the potential off-target pharmacological effects of drugs inhibiting autophagy, we treated cells with small interfering RNAs against ATG5 or ATG7, two essential components for the formation of autophagosome. As shown in Fig. 5D and Fig. S2, siRNA against ATG5 or ATG7 could efficiently knockdown its target gene, and blocked linifanib-induced autophagy in Bel-7404 cells. Similar to the results of autophagy inhibitors, siRNA against ATG5 or ATG7 significantly enhanced the cytotoxicity of linifanib in Bel-7404 cells by promoting apoptosis (Fig. 5E, F & G).

Generally, these data demonstrated that linifanib-induced autophagy was cytoprotective. Inhibition of autophagy could enhance the growth inhibitory effect of linifanib, at least partially, through promotion of apoptosis in HCC cells.

### Autophagy inhibition enhances the anti-tumor effect of linifanib *in vivo*

**Figure 6:**
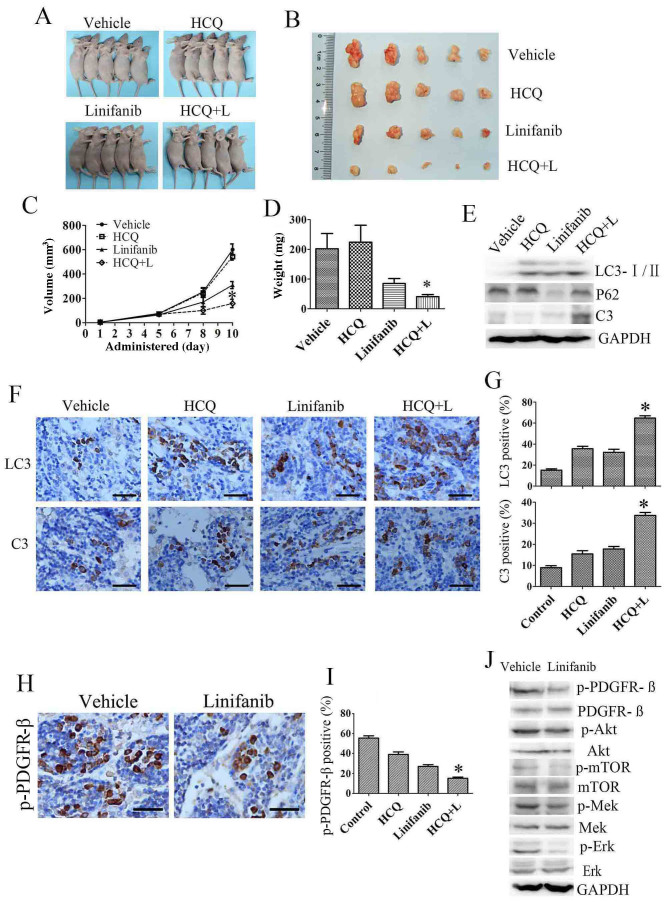
Autophagy inhibition enhances the anti-tumor effect of linifanib in Bel-7404 xenograft models. (A&B). Tumors from nude mice in each treatment group. (C&D). Tumor volume and weight in each group. (E). Tumor lysate was subjected to immunoblotting for LC3, p62 and cleaved caspase-3 (C3). Each lane represents lysate from five mice. (F). Immunohistochemical staining of LC3 and cleaved caspase-3 using paraffin embedded sections. (G). Quantification of LC3, cleaved caspase-3 staining by Image Pro Plus 5.0. (H). Immunohistochemical staining of p-PDGFR-ß in vehicle or linifanib treated tumor. (I). Quantification of the p-PDGFR-ß by Image Pro Plus 5.0. (J). Tumor lysate was subjected to immunoblotting for PDGFR-ß and down-stream Akt/mTOR and Mek/Erk signaling pathways. Each lane represents lysate from five mice. Data were expressed as the mean ± SD. C, D, G & I, Linifanib + HCQ *vs* linifanib, * P<0.05.

To further determine the therapeutic benefit of inhibiting autophagy in combination with linifanib, we used HCC xenograft model generated by grafting the Bel-7404 cells. When the xenografted tumors were measurable, mice were randomly assigned to receive HCQ, linifanib or a combination of HCQ with linifanib. As shown in Fig. 6A–D, HCQ alone had no effect on the growth of tumors, and linifanib alone displayed a moderate anti-tumor activity. In contrast, combination of HCQ with linifanib significantly reduced tumor growth compared to the linifanib alone. Furthermore, no significant weight loss was observed by combination treatments (data not shown).

We then investigated the effect of linifanib with or without HCQ on autophagic and apoptotic pathways in xenografted tumors. As shown in Fig. 6E, Tumors treated with linifanib displayed accumulation of LC3-II. In addition, the expression of LC3-II is much higher in linifanib plus HCQ group than that in linifanib or HCQ alone. In parallel to this LC3-II increase, a drastic degradation of autophagy substrate p62 in tumor tissues was observed after linifanib treatment. However, HCQ slightly increased p62 expression compared to the control group. Combination with HCQ partially blocked the linifanib-induced p62 degradation. Moreover, Caspase-3 is significantly activated in linifanib plus HCQ group, indicating that blockage of autophagy enhanced linifanib-induced apoptosis of tumor cells. Immunohistochemical analysis re-confirmed that linifanib induced autophagy in xenografted tumor, and inhibition of autophagy promoted apoptosis, as reflected by LC3 and cleaved caspase 3 staining (Fig. 6F&G). We next investigated the mechanism of linifanib-induced autophagy *in vivo*. Consistence with the *in vitro* results, linifanib induced autophagy through inhibition of phospho-PDGFR-β and its downstream pathways, including Akt/mTOR and Mek/Erk signaling pathways. (Fig. 6H, I& J).

Taken together, these findings suggest that linifanib induces HCC cells autophagy *in vivo* through inhibiting the phosphorylation of PDGFR-β and its down-stream Akt/mTOR and Mek/Erk signaling pathways. Inhibition of autophagy by HCQ can potently enhance the anti-HCC activity of linifanib *in vivo*.

## Discussion

Autophagy constitutes a potential target for cancer therapy. A number of studies have shown that tyrosine kinase inhibitors (TKIs) and antibodies can activate autophagy in cancer cells, such as imatinib^29^,^30^, sorafenib^15^,^31^,^32^, crizotinib^33^, pazopanib^34^,^35^, sunitinib^35^,^36^, and cetuximab^16^,^37^ etc. In our previous study, we reported that EGFR tyrosine kinase inhibitors gefitinib and erlotinib activate autophagy in human lung cancer cells^17^. Induction of autophagy in response to these TKIs can be viewed as having a prodeath or a prosurvival role, which contributes to the anticancer efficacy of these drugs as well as drug resistance. The exact function of autophagy in TKIs-treated cancer cells is context dependent. In this study we investigated the role of autophagy in HCC cells treated with a novel multikinase inhibitor linifanib. We observed the shift of LC3-I to LC3-II in HCC cells after linifanib exposure (Fig. 1A & B). Immunofluorescence also showed that the punctate endogenous LC3 was increased in linifanib treated cells (Fig. 1C & D). A classic autophagy detection method, TEM, also showed that autophagosome was significantly increased in cells treated with linifanib (Fig. 2). These results provided the strong evidences that linifanib activated autophagy in HCC cells. However, autophagy was a highly dynamic and multi-step process. Thus, these results should be interpreted carefully because upregulation of LC3-II or formation of autophagosomes may reflect either the induction of autophagy or reduction of autophagic turnover. In order to distinguish these two possibilities, we used a functional screening approach to evaluate the autophagic flux^22^, As shown in Fig. 3D, the ratio of LC3^WT^/LC3^G120A^ was decreased significantly when cells were treated with 2.5 μM linifanib for 24 or 48 hours, indicating that linifanib accelerated autophagic flux and stimulated the degradation of LC3. All these results together thus suggest that linifanib activates autophagy in HCC cells.

PDGF and its receptors play crucial roles in the development and maintenance of liver tumor^38^. The levels of both PDGFR α and ß represent valuable prognostic markers in patients with HCC^39^. In this study, we found PDGFR-ß, but not PDGFR-α, was overexpressed in HCC cells (data not show). Knockdown of PDGFR-ß by siRNA led to upregulation of LC3-II and downregulation of p62, indicating that knockdown of PDGFR-ß could activate autophagy. Akt/mTOR and Mek/Erk pathways are two major downstream pathways of PDGFR-ß. The inhibitory effect of linifanib on the Erk and Akt phosphorylation was reported in Ewing sarcoma cells and acute myeloid leukemia cells^8^,^9^. However, the precise effect of linifanib on Akt/mTOR pathway and Mek/Erk signaling in HCC cells has not been established clearly. Akt/mTOR and Mek/Erk pathways are not only two critical signaling pathways involved in regulating cell proliferation, migration, differentiation and death, but also play important roles in regulating autophagy^27^. For example, sorafenib and cetuximab induced autophagy by inhibition of Akt/mTOR signaling^15^,^16^. Daunorubicin induces cytoprotective autophagy by activation of ERK in myeloid leukemia cells^40^. Thus, we analyzed phosphorylatory levels of Akt/mTOR/p70S6 and Mek/Erk in HCC cells treated with linifanib. Our results clearly demonstrated that linifanib could inhibit the phosphorylation of Akt/mTOR and Mek/Erk. Because inhibition of mTOR results in activation of autophagy, we speculate that linifanib activates autophagy via inhibiting PDGFR-ß and its downstream Akt/mTOR pathway. To our best knowledge, this is the first report to document that autophagy can be activated in HCC cells by inhibition of PDGFR-ß and its downstream pathways.

Wang J, *et al* reported that activation of Mek/Erk signaling in tumor cells could activate autophagy^41^. In our previous study, we also found that daunorubicin induced autophagy by activation of Mek/Erk pathway in human myeloid leukemia cell line K562^40^. However, in this study, we found that linifanib inhibited the phosphorylation of Mek/Erk and induced autophagy. How to define the role of Mek/Erk in linifanib-induced autophagy? One possible explanation is that Mek/Erk signaling does not play a critical role in autophagy activated by linifanib. The other explanation is that inhibition of Mek/Erk can also activate autophagy in HCC cells. To evaluate whether inhibition of Mek/Erk signaling can activate autophagy, we used Mek inhibitor U0126 to treat Bel-7404 cells. As shown in Fig. S3, treatment with U0126 led to inhibition of Erk, upregulation of LC3-II and downregulation of p62. This result clearly demonstrated that inhibition of Mek/Erk pathway in Bel-7404 could activate autophagy. In light of these observations, we propose that linifanib inhibits PDGFR-ß and its downstream Akt/mTOR and Mek/Erk signal pathways, and subsequently triggers autophagy in hepatocarcinoma cells.

Another interesting finding in this study is that linifanib treatment appears to induce lysosomal accumulation (Fig 1C and Supplementary Fig 1&2). The LysoTracker probes are fluorescent acidotropic probes for labeling and tracking acidic organelles (mainly lysosomes) in live cells. Lysosomes are dynamic organelles and are involved in late digestive stages of autophagy. Some literatures indicate that lysosomal mass increases during the autophagic process to enable the cell to produce autolysosomes^42^,^43^. Zhou et al reported that suppression of mammalian target of rapamycin (mTOR) activity by starvation or mTOR catalytic inhibitors promoted activation of lysosomal function^44^. They provided strong evidences that the functional activation of lysosome in the course of autophagy is via suppression of mTORC1 and autophagosome-lysosome fusion. ATG5 or ATG7 deletion or blockage of the autophagosome-lysosome fusion process effectively diminished lysosomal activation^44^. In our study, we also found that linifanib inhibited mTOR phosphorylation. Thus, we proposed that the phenomenon of lysosome accumulation accompanied with enhanced autophagy was caused by mTOR inhibition in linifanib-treated cells. And siRNA knockdown of ATG5 or ATG7 blocked linifanib-induced lysosome activation (Fig.1C and supplementary Fig. 2). However, the detailed molecular mechanism by which linifanib induces lysosome accumulation needs further investigation.

As we mentioned above that normal cells also need autophagy to maintain homeostasis, one should be noted that inhibition of autophagy increased the susceptibility to chemotherapy in normal cells. Takahashi and colleagues found that kidney injury was increased in autophagy deficient mice, compared to wild-type mice after cisplatin administration^45^. Periyasamy-Thandavan and colleagues observed that autophagy was cytoprotective during cisplatin injury of renal proximal tubular cells^46^. As an antimalarial medicine, HCQ has a long history without significant toxicity. In our xenograft models, we didn't find any abnormal symptoms in mice treated with HCQ plus linifanib, such as hematuria, which was correlated with acute kidney injury. However, it is not clear whether chronic kidney or liver injury would occur after linifanib plus HCQ treatment because the exposure time in this study is only 10 days. Thus, in future studies, we may focus on the effect of autophagy inhibitor combined with linifanib on normal cells, such as renal proximal tubular cells and normal liver cells, in a condition of prolonged exposure time.

In summary, we conclude that a decrease in Akt/mTOR and Mek/Erk signaling resulting from PDGFR-ß inhibition by linifanib can activate autophagy in HCC cells, which contributes to the survival of HCC cells both *in vitro* and *in vivo*. Inhibition of autophagy by pharmacological or genetic approaches sensitized HCC cells to linifanib, at least partially, through promoting linifanib-induced apoptosis. Autophagy inhibition represents a promising approach to surmount intrinsic or acquired resistance to linifanib treatment. Thus, linifanib combined with autophagy inhibitors could be translated into clinical practice in the future.

## Methods

### Reagents and antibodies

The chemicals used were linifanib (Cayman, Michigan, USA), chloroquine (CQ), hydroxychloroquine (HCQ) (J&K chemical Ltd, Beijing, China) and 3-methyladenine (3-MA) (Sigma-Aldric*h* Corporation, St Louis, MO, USA). The primary antibodies against microtubule-associated protein 1 light chain 3 (LC-3), ATG5, p62, ATG7, Beclin-1, Caspase-3, total or phospho-Akt, mTOR, S6K, Mek, Erk and PDGFR-ß were from Cell signaling technology (Cell Signaling Technology, Boston, MA, USA). The secondary antibodies were HRP conjugated anti-rabbit, anti-mouse IgG (Cell Signaling Technology) or FITC conjugated anti-rabbit IgG (Beyotime, Nanjing, China).

### Cell lines and animals

HepG2 and Bel-7404 cells were purchased from cell bank of Chinese Academy of Science. Cells were maintained in DMEM (Gibco, Carlsbad, CA) supplemented with 10% fetal bovine serum (Gibco). Linifanib was dissolved in dimethyl-sulphoxide (DMSO) and was further diluted with medium before use. Final concentration of DMSO was 0.1%. Female athymic BALB/c nude mice (Shanghai Institute of Material Medicine, Chinese Academy of Science, China) were maintained in a specific pathogen-free facility and treated with humane care with approval from the Animal Care and Use Committee of Zhejiang University.

### Proliferation assay

Cell proliferation was determined by MTS assay. Cells were seeded into 96-well plates and treated with linifanib, CQ, 3-MA or combination. After treatment, 10 μl MTS (Promega, Madison, WI, USA) was added into each well for 2-h incubation. The absorbance was measured using a model ELX800 Micro Plate Reader (Bio-Tek Instruments, Inc, Winooski, USA) at 490 nm then calculate the proliferation.

### Transmission electron microscopy

Treated cells were washed and fixed for 30 min in 2.5% glutaraldehyde. The samples were then treated with 1.5% osmium tetroxide, dehydrated with acetone and embedded in Durcupan resin. Thin sections were poststained with lead citrate and examined in the TECNAI 10 electron microscope (Philips, Eindhoven, Netherlands) at 60 kV.

### Immunofluorescence

Cells, seeded at 3 × 10^5^ into six-well plate containing glass, were treated with designed dose of chemicals for 24-h, then were incubated with Lyso Tracker (Invitrogen, Carlsbad, CA, USA) for 90 min. After this, cells were washed twice with PBS, followed by fixation in 4% paraformaldehyde and permeabilized with 1% CHAPS buffer (150 mM NaCl, 10 mM HEPES, 1.0% CHAPS) at room temperature for 15 min. Hereafter, cells were incubated with anti-LC3 (Sigma-Aldrich, L7543) for 2-h at 37°C, then incubated with FITC-conjugated anti-rabbit IgG for 1-h at 37°C. After that, cell nuclei were stained by DAPI (Invitrogen) for 15 min. Samples were examined under a Zeiss LSM 710 fluorescence microscope system (Carl Zeiss Inc, Oberkochen, Germany). Image was processed with ZEN LE software. For quantification of LC3-positive cells, 150–200 cells were randomly selected from the acquired image and counted. The cells with more than five dots of specific green or yellow signals were considered to be LC3-positive.

### RLuc-based Screening Assay

Renilla luciferase (RLuc)–LC3^WT^ and RLuc–LC3^G120A^ plasmids were kindly provided by Dr. Marja Jäättelä. Transfection was performed by attractene (Qiagen, Valencia, CA, USA) according to the manufacturer's instructions. Stable transformants were selected in a complete medium containing 500 μg/ml G418 (Sigma-Aldrich). Stable expression of RLuc–LC3^WT^ and RLuc–LC3^G120A^ of Bel-7404 cells were seeded side by side in 96-well plate and treated with various concentration of linifanib. 22 hours after treatment, 50 nM of Enduren substrate (Promega) was added. RLuc activity was measured at 24 and 48 h after linifanib treatment. Luciferase measurements were done using the DTX 800 Multimode Detector (Beckman Coulter, Fullerton, CA, USA).

### RNA interference

Cells were transfected with antisense or siRNA against ATG5 siRNA (Qiagen, SI02655310), ATG7 siRNA (Qiagen, SI02655373) or non-specific siRNA (Qiagen, 1027280) using Lipofectamine 2000 (Invitrogen) according to manufacturer's protocol. For PDGFR-β RNA interference, two siRNA oligonucleotides targeting PDGFR-β were synthesized by GenePharma (Shanghai, China). The sequences of the sense strands of the RNAs targeting PDGFR-β used in this study were as follows: PDGFR-β siRNA 1 GUGAGAAGCAAGCCCUUAUTT, PDGFR-β siRNA 2 CUCCAGUGCUAAGCUACAUTT. A nonspecific oligo that is not complementary to any human genes was used as a negative control.

### Western blot analysis

Cells were lysed and immunoblotted as previously described^47^. Briefly, proteins were resolved by SDS-polyacrylamide gel electrophoresis, transferred to a PVDF membrane (Millipore, Billerica, MA, USA) and then detected by the proper primary and secondary antibodies before visualization with a chemiluminescence kits (Biological Industries, Kibbutz Beth HaEmek, Israel). Visualization was done with Image Quant LAS-4000 (Fujifilm, Tokyo, Japan) using image Multi-Gauge Software (Fujifilm).

### Apoptosis assay

Cell apoptotic rate was determined by flow cytometry analysis with the fluorescein isothiocyanate (FITC) Annexin V Apoptosis Detection Kits (KeyGEN Biotech, Nanjing, China). Cells were collected by trypsinization, washed twice and resuspended in 1 × binding buffer at a concentration of 1 × 10^6^ cells/ml. Then mixed 100 μl of cells with 5 μl of FITC Annexin V and 5 μl PI, and incubated for 15 min. The samples were then sent out for analysis by flow cytometry. The results were analyzed with the BD FACSCalibur™ system.

### Human HCC xenograft model

To establish Bel-7404 tumors, 5 × 10^6^ cells were inoculated *s.c.* in the flank region of 5–6 week-old female athymic BALB/c nude mice. When the diameter of the subcutaneous tumor reached about 0.5 centimeter, tumor-bearing animals were randomly assigned to vehicle, linifanib alone, HCQ alone or linifanib + HCQ. Linifanib was suspended in corn oil and administered by oral gavage at the dose of 10 mg/kg/day. HCQ was dissolved in 0.9% NaCl and intraperitoneally administered daily at the dose of 60 mg/kg/day. Tumor volume was calculated using the formula V = AB^2^/2, where A is the largest diameter and B is the smallest diameter. Mice were sacrificed 24 hours after the last treatment. The tumors were weighed and subjected for western blot or paraffin embedding. Immunohistochemistry was performed on formalin-fixed and paraffin-embedded 4 μm sections of tumor samples using adequate primary antibodies. Images were visualized using a Zeiss LSM 710 fluorescence microscope system (Carl Zeiss Inc). Image was processed with ZEN LE software.

### Ethics statement

The animal study is approved by the institutional animal ethical committee of Zhejiang University with approval No. zju-2012-1-01-088. The methods were carried out in accordance to the approved guidelines.

### Statistical analysis

Unless otherwise stated, data were expressed as the mean ± SD, and analyzed by Student's t test. P < 0.05 was considered statistically significant.

## Author Contributions

W.H., W.H. and X.C. conceived the concept and designed the study; H.P., Z.W., L.J., X.S., L.Y., J.S., Z.J., J.X. and W.G. performed the experiments; H.P. and W.H. analyzed the data and prepared the figures; W.H. and Z.W. wrote the paper; All authors reviewed the manuscript.

## Supplementary Material

Supplementary Information Supplementary materials
