# Author Correction: Autophagy inhibition sensitizes hepatocellular carcinoma to the multikinase inhibitor linifanib

**DOI:** 10.1038/s41598-020-74205-7

**Published:** 2020-10-07

**Authors:** Hongming Pan, Zhanggui Wang, Liming Jiang, Xinbing Sui, Liangkun You, Jiawei Shou, Zhao Jing, Jiansheng Xie, Weiting Ge, Xiujun Cai, Wendong Huang, Weidong Han

**Affiliations:** 1 Department of Medical Oncology, Sir Run Run Shaw Hospital, College of Medicine, Zhejiang University, Zhejiang, Hangzhou China; 2 Laboratory of Cancer Biology, Zhejiang, Hangzhou China; 3 Cancer Institute, The Second Affiliated Hospital, College of Medicine, Zhejiang University, Zhejiang, Hangzhou, China; 4 Department of General Surgery, Sir Run Run Shaw Hospital, College of Medicine, Zhejiang University, Zhejiang, Hangzhou China; 5 Division of Molecular Diabetes Research, Department of Diabetes and Metabolic Diseases Research, Beckman Research Institute, City of Hope National Medical Center, Duarte, CA USA; 6 Institute of Clinical Science, Sir Run Run Shaw Hospital, College of Medicine, Zhejiang University, Zhejiang, Hangzhou China

Correction to: *Scientific Reports* 10.1038/srep06683, published online 20 October 2014

This Article contains errors in Supplementary Figure 1, where the images of Linifanib and 3-MA + Linifanib are duplications of the images of Linifanib and siATG5 + Linifanib in Supplementary Figure 2A, respectively. The correct Supplementary Figure 1 appears here as Figure 1.

**Figure 1:**
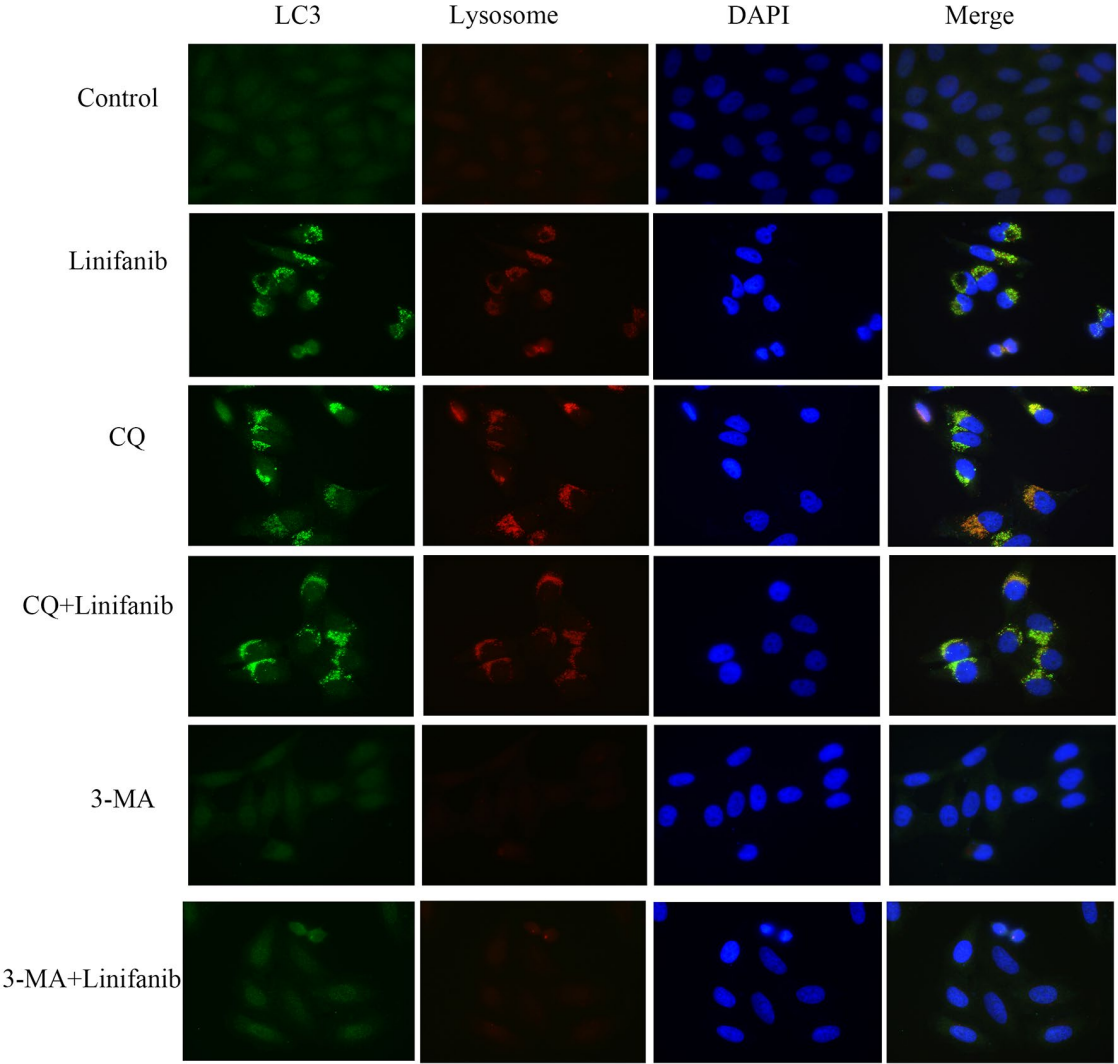
Linifanib induced autophagosomes and autolysosomes formation in hepatocarcinoma cells. Bel-7404 cells were treated with 2.5 μM linifanib in the presence or absence of CQ (5 μM) or 3-MA (5 mM) for 24 h before labeled with fluorescence and imaged by fluorescence microscope. Green: FITC-labeled LC3; Red: lyso-tracker-labeled lysosome; Blue: DAPI-labeled nucleus. The yellow puncta indicated LC3 co-located with lysosome.

