# Supplementary material for: Autophagy inhibition sensitizes hepatocellular carcinoma to the multikinase inhibitor linifanib
Source: Sci Rep. 2014 Oct 20;4:6683. doi: 10.1038/srep06683 (PMC4202209; doi:10.1038/srep06683)
Supplement: Supplementary Information — Supplementary materials [file srep06683-s1.pdf]

Supplementary materials

Autophagy inhibition sensitizes hepatocellular carcinoma to the multikinase inhibitor linifanib

Hongming Pan[1]*, Zhanggui Wang[1]*, Liming Jiang[1], Xinbing Sui[1], Liangkun You[1], Jiawei Shou[1], Zhao Jing[1], Jiansheng Xie[2], Weiting Ge[3], Xiujun Cai[4#], Wendong Huang[5#], Weidong Han[1#]

[1]Department of Medical Oncology, [2]Laboratory of Cancer Biology; Institute of Clinical Science, [4]Department of General Surgery, Sir Run Run Shaw Hospital, College of Medicine, Zhejiang University, Hangzhou, Zhejiang, China; [3]Cancer Institute, The Second Affiliated Hospital, College of Medicine, Zhejiang University, Hangzhou, Zhejiang, China. [5]Division of Molecular Diabetes Research, Department of Diabetes and Metabolic Diseases Research, Beckman Research Institute, City of Hope National Medical Center, Duarte, CA.

* These authors contributed equally to this work.

Corresponding authors:

Weidong Han, Department of Medical Oncology, Sir Run Run Shaw Hospital, College of Medicine, Zhejiang University, 3 East Qingchun Road, Hangzhou 310016, China. Tel.: +86 571 86006926; Fax: +86 571 86044817; E-mail: hanwd@zju.edu.cn;

Xiujun Cai, Department of General Surgery, Sir Run Run Shaw Hospital, College of Medicine, Zhejiang University, 3 East Qingchun Road, Hangzhou 310016, China. Tel.: +86 571 86006926; Fax: +86 571 86044817; E-mail: xjcaizju@163.com;

Wendong Huang, Division of Molecular Diabetes Research, Department of Diabetes and Metabolic Diseases Research, Beckman Research Institute, City of Hope National Medical Center, 1500 E. Duarte Road, Duarte, CA 91010. Tel.: 626-256-4673; Fax:626-256-8704; E-mail: whuang@coh.org.

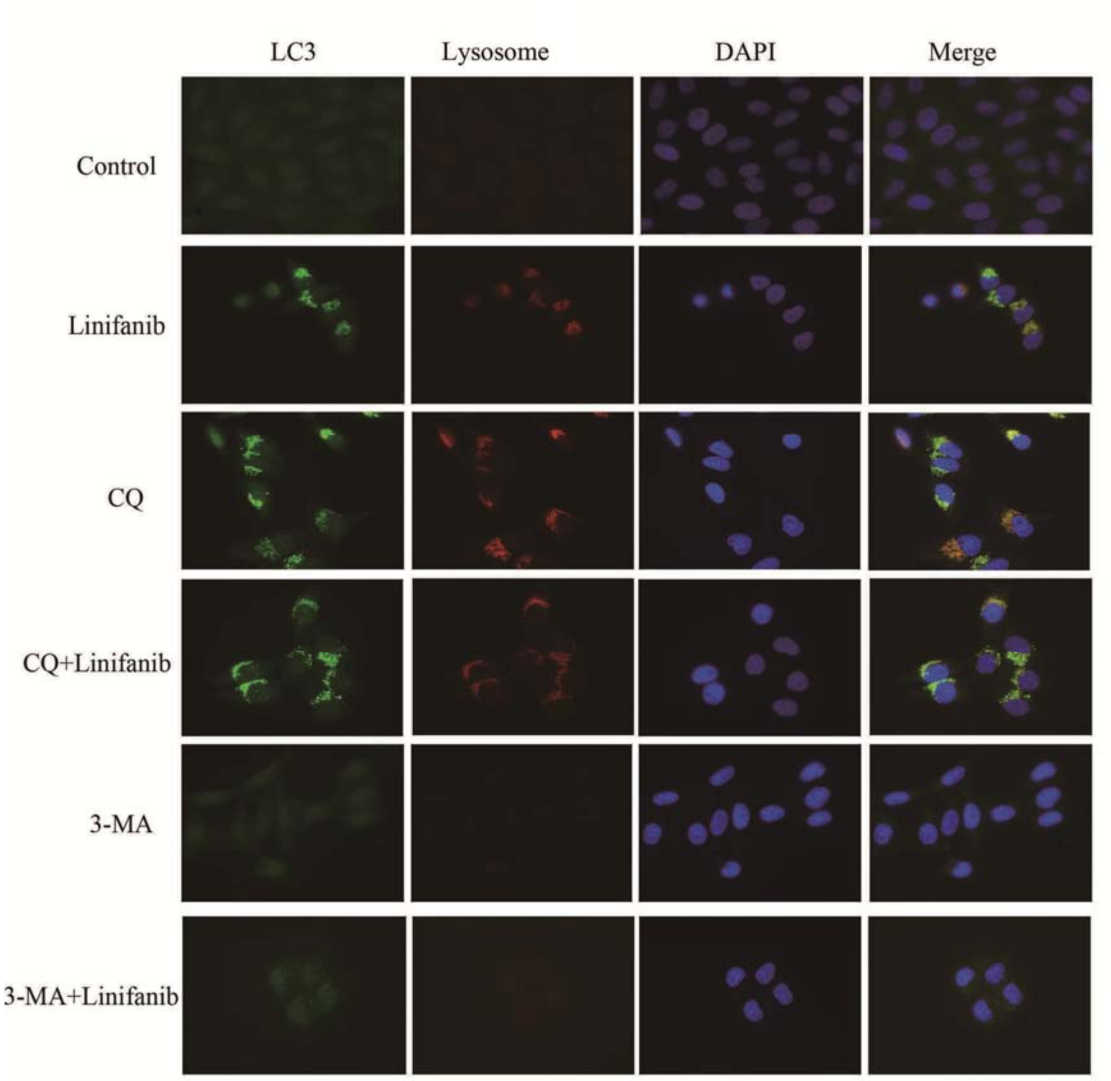

Fig. S1. Linifanib induced autophagosomes and autolysosomes formation in hepatocarcinoma cells. Bel-7404 cells were treated with 2.5 μM linifanib in the presence or absence of CQ (5 μM) or 3-MA (5 mM) for 24 h before labeled with fluorescence and imaged by fluorescence microscope. Green: FITC-labeled LC3; Red: lyso-tracker-labeled lysosome; Blue: DAPI-labeled nucleus. The yellow puncta indicated LC3 co-located with lysosome.

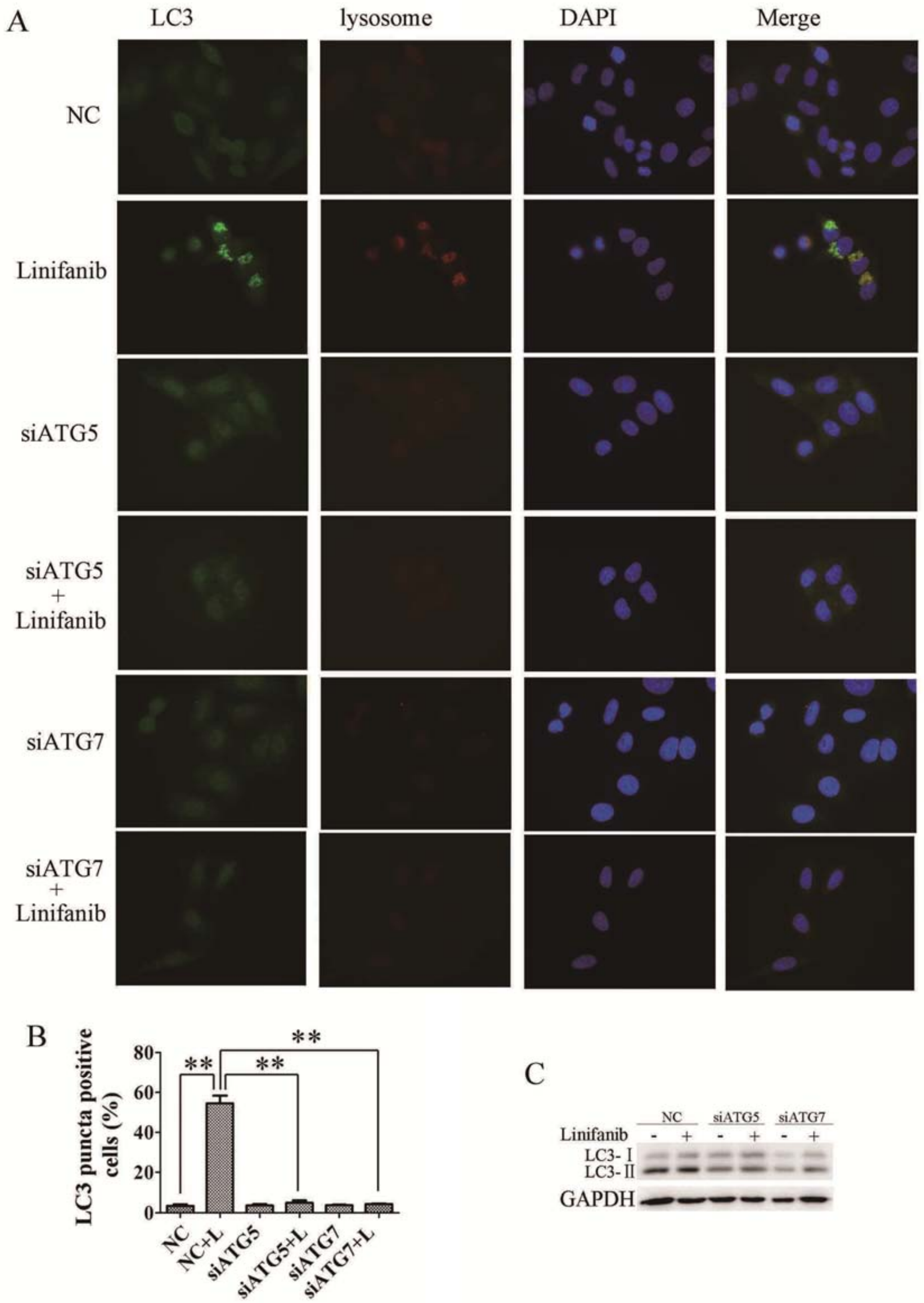

Fig. S2. Inhibition of linifanib-induced autophagy by siRNAs against ATG5 or ATG7. Bel-7404 cells were transfected with siRNA against ATG5 (siATG5) or ATG7 (siATG7) for 24 h, then treated with linifanib (L) for 24 h. A. Cells were labeled with fluorescence and imaged by fluorescence microscope. Green: FITC-labeled LC3; Red:

lyso-tracker-labeled lysosome; Blue: DAPI-labeled nucleus. B. Percentage of green or yellow puncta-positive cells was quantified and analyzed using a threshold of >5 dots/cell. The data were represented as the mean ± SD. **P < 0.01. C. Cell lysate was subjected to immunoblotting for LC3. Data were expressed as the mean±SD. ** P<0.01.

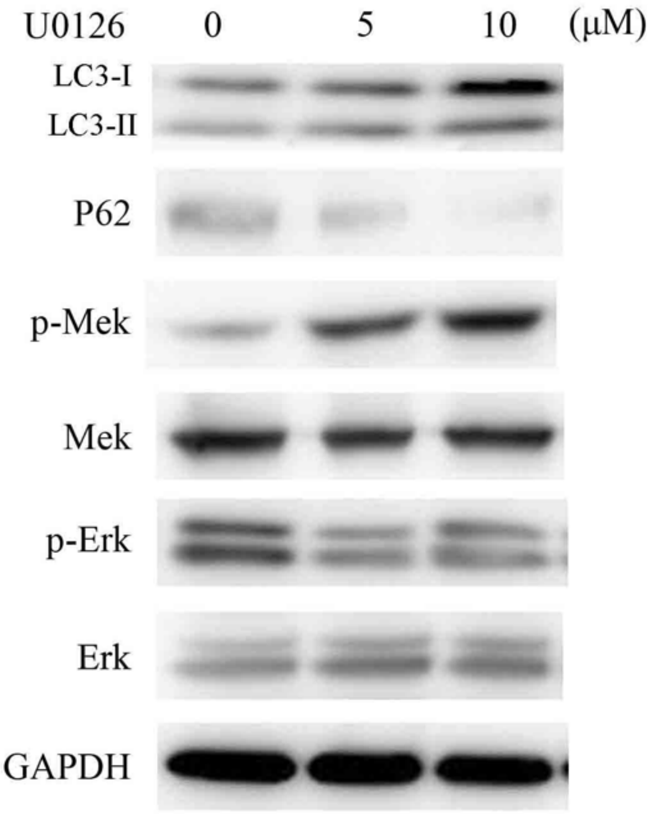

Fig. S3. The Mek/Erk-selective inhibitor U0126 induces autophagy in Bel-7404 cells. Cells were treated with U0126 for 24 hours. Cell lysates were subjected to immunoblotting analysis for LC3, p62, and phospho- or total forms of Mek, Erk.
